# Xeno-Nucleic Acid (XNA) 2’-Fluoro-Arabino Nucleic Acid (FANA) Aptamers to the Receptor-Binding Domain of SARS-CoV-2 S Protein Block ACE2 Binding

**DOI:** 10.3390/v13101983

**Published:** 2021-10-02

**Authors:** Irani Alves Ferreira-Bravo, Jeffrey J. DeStefano

**Affiliations:** 1 Cell Biology and Molecular Genetics, Bioscience Research Building, University of Maryland, College Park, MD 20742, USA; bioirani@gmail.com; 2 Maryland Pathogen Research Institute (MPRI), College Park, MD 20742, USA

**Keywords:** aptamer, XNA, FANA, SARS-CoV-2 RBD, ACE2

## Abstract

The causative agent of COVID-19, SARS-CoV-2, gains access to cells through interactions of the receptor-binding domain (RBD) on the viral S protein with angiotensin-converting enzyme 2 (ACE2) on the surface of human host cells. Systematic evolution of ligands by exponential enrichment (SELEX) was used to generate aptamers (nucleic acids selected for high binding affinity to a target) to the RBD made from 2ʹ-fluoro-arabinonucleic acid (FANA). The best selected ~79 nucleotide aptamers bound the RBD (Arg319-Phe541) and the larger S1 domain (Val16-Arg685) of the 1272 amino acid S protein with equilibrium dissociation constants (*K*_D,app_) of ~10–20 nM, and binding half-life for the RBD, S1 domain, and full trimeric S protein of 53 ± 18, 76 ± 5, and 127 ± 7 min, respectively. Aptamers inhibited the binding of the RBD to ACE2 in an ELISA assay. Inhibition, on a per weight basis, was similar to neutralizing antibodies that were specific for RBD. Aptamers demonstrated high specificity, binding with about 10-fold lower affinity to the related S1 domain from the original SARS virus, which also binds to ACE2. Overall, FANA aptamers show affinities comparable to previous DNA aptamers to RBD and S1 protein and directly block receptor interactions while using an alternative Xeno-nucleic acid (XNA) platform.

## 1. Introduction

The coronavirus SARS-CoV-2 has had a devastating impact on society that will likely continue into the foreseeable future. It is the third coronavirus (SARS-CoV-1 and MERS being the other two) to emerge as a human pathogen in the past 17 years, raising the possibility that others will arise in the future [1,2]. Thus, the development of novel therapeutics targeting SARS-CoV-2 and new approaches that can potentially be extended to emerging or future viruses are urgently needed. Infection with SARS-CoV-2 requires interaction between the viral surface protein, spike (S), and a host “receptor” protein, angiotensin-converting enzyme 2 (ACE2) [3], that is expressed on type II alveolar cells [4] and ciliated cells in the human airway epithelium (HAE) [5], making these cells potentially vulnerable to infection. Antibodies that block this interaction have been successfully used to mitigate COVID-2 infections [6,7,8].

In this report, we describe the selection of aptamers, which are short nucleic acid-based sequences that bind with high affinity to targets, to block the interaction between the S protein and the ACE2 receptor. Aptamers have many applications including the replacement of antibodies in biochemical assays (e.g., ELISA), utilization as biosensors (including a recent rapid test for SARS-CoV-2 [9]), and as tools for studying virus molecular biology, and development of antiviral drugs [10,11,12,13,14,15,16,17,18]. Aptamers have shown potent antiviral activity and low toxicity in cell culture [14,19,20,21,22,23,24,25,26,27,28,29], and they are among the most potent inhibitors of protein activity in vitro [30,31,32]. 

Aptamers are typically made from natural RNA or DNA using systematic evolution of ligands by exponential enrichment (SELEX) [33,34]. More recently, xeno-nucleic acids (XNA), which are nucleotide analogs with altered sugar, base, or phosphate backbones, have been employed in place of DNA or RNA. Aptamers offer strong promise as therapeutics and diagnostics, as they have low immunogenicity and, in the case of XNA aptamers, greater resistance to degradation [35,36,37,38,39]. Our group was the first to produce 2’-fluoro-arabino nucleic acid (FANA) [40] XNA aptamers to proteins [41,42]. These aptamers bind with exceptionally high affinity to targets and are completely RNase resistant. In this report, we describe the generation of FANA aptamers to the receptor-binding domain (RBD) of the SARS-CoV-2 S protein that can block the interactions between the S protein and ACE2 receptor. Although this work is targeted for SARS-CoV-2, the established principles could potentially be used for other current or future viruses and the discovered aptamers have potential as virus inhibitors, but also in diagnostics and as biosensors. 

## 2. Materials and Methods

### 2.1. Materials

The 2′-deoxy-2′-fluoro-arabino nucleotides (faATP, faCTP, faGTP, faUTP) required for FANA synthesis were obtained from Metkinen Chemistry (Kuusisto, Finland). Deoxyribonucleotide triphosphates (dNTPs) were from Roche (Penzberg, Germany) or United States Biochemical (Cleveland, OH). Enzymes and buffers including *Taq* polymerase, T4 polynucleotide kinase (PNK), 10X ThermoPol buffer (Mg^2+^-free), and MgSO_4_ were from New England BioLabs (Ipswich, MA). Radiolabeled ATP (γ-^32^P) was from PerkinElmer^®^ (Waltham, MA). G-25 spin columns were from Harvard Apparatus (Holliston, MA). Miniprep DNA preparation kits were purchased from QIAGEN (Hilden, Germany). Nitrocellulose filter disks (Protran BA 85, 0.45 μm pore size and 25 mm diameter) were from Whatman (Maidstone, United Kingdom). Magnetic beads (Dynabeads™ His-Tag Isolation and Pulldown) for selection were from Invitrogen (Waltham, MA). All DNA oligonucleotides were from Integrated DNA Technologies (IDT) (Coralville, IA). Thermostable polymerase D4K for FANA nucleic acid production was prepared as described and stored in aliquots at −80 °C [36]. The SARS-CoV-2 receptor binding domain (RBD) (Arg319-Phe541) (Wuhan strain) and human ACE2 protein (both C-terminal His-tagged) were from RayBiotech (Peachtree Corners, GA). The C-terminal His-tagged SARS-CoV-1 S1 protein (Met1-Arg667) was from SinoBiological (Beijing, China). The His-tagged (Val16-Arg685) and untagged (Gln14-Arg685) SARS-CoV-2 S1 proteins (both Wuhan strain), cPass™ SARS-CoV-2 Neutralization Antibody Detection kit, and monoclonal antibody (clone ID: 6D11F2) were from GenScript^®^ (Piscataway, NJ). The C-terminal His-tagged SARS-CoV-2 S protein trimer construct (Wuhan strain) was from Antibodies-online Inc (Davis, CA). The C-terminal His-tagged RBD Delta variant (Lys452Arg, Thr478Lys, compared with Wuhan strain RBD) protein was from Abbexa (Cambridge, United Kingdom). All other chemicals were from Avantor (Radnor, PA), Fisher Scientific (Waltham, MA), or Sigma (St. Louis, MO).

### 2.2. Methods

#### 2.2.1. End-Labeling of Oligonucleotides with T4 Polynucleotide Kinase 

DNA oligonucleotides were 5′ end-labeled in a 50 μL volume containing 10–250 pmol of the oligonucleotide of interest, 1X T4 PNK reaction buffer (provided by the manufacturer), 10 U of T4 PNK and 5–10 μL of (γ-^32^P) ATP (3000 Ci/mmol, 10 μCi/μL). The labeling reaction was performed at 37 °C for 30 min according to the manufacturer’s protocol. PNK enzyme was heat inactivated by incubating the reaction at 75 °C for 15 min. Excess radiolabeled nucleotides were then removed by centrifugation using a Sephadex G-25 column.

#### 2.2.2. Selection of FANA Aptamers with SARS-CoV-2 RBD Using Magnetic Dynabeads™ 

The 79-nucleotide FANA random pool starting material (referred to as FANA-ST) for SELEX containing a 40-nucleotide central random region flanked at the 5′ end by 20 nucleotides of fixed sequence DNA (5′-AAAAGGTAGTGCTGAATTCG-3′), and at the 3′ end by 19 nucleotides of fixed FANA sequence (5′-UUCGCUAUCCAGUUGGCCU-3’) (i.e., 5′-AAAAGGTAGTGCTGAATTCG(N)_40_UUCGCUAUCCAGUUGGCCU-3’), was prepared as described previously [41]. About 200 pmol (~1 × 10^14^ different sequences) of 5′ ^32^P-labeled FANA starting pool was heated to 90 °C then snap-cooled on ice. The material was then incubated with 20 pmoles of SARS-CoV-2 RBD protein that had been attached to Dynabeads™ using the C-terminal His-tag. Incubations were in 200 µL PBS (137 mM NaCl, 2.7 mM KCl, 8 mM Na_2_HPO_4_, and 2 mM KH_2_PO_4_, pH 7.4) for 30 min with agitation at room temperature. The beads were washed 2× with 200 µL of PBS and the bound FANA material was removed by adding 200 µL of imidazole containing buffer (300 mM imidazole, 50 mM sodium phosphate pH 8.0, 300 mM NaCl, 0.01% Tween™-20) to the beads and heating for 5 min at 90 °C, then removing the beads with a magnet. Bound FANA was recovered by precipitation with ethanol in the presence of 50 μg of glycogen. The material was reverse transcribed to DNA, amplified and converted to FANA for another round of selection as previously described [41]. The SELEX was stopped after round 8 as no further binding affinity increase was detected. 

#### 2.2.3. Sequence Analysis of FANA Products Recovered from Round 8 

PCR products were prepared from FANA sequences recovered from round 8. The PCR material was cloned using a TOPO TA cloning kit from Life Technologies. DNA mini-preps were prepared, and the products were sequenced by Macrogen (Rockville, Maryland). The appropriate DNA oligonucleotide templates for some of the recovered sequences were synthesized, and generation of FANA material was performed as described [41].

#### 2.2.4. Determination of Apparent Equilibrium Dissociation Constant (*K*_D,app_) Using Nitrocellulose Filter Binding Assays

Standard reactions for *K*_D,app_ determinations were performed in 20 µL of PBS with 0.1 mg/mL BSA and 0.1 nM 5′ ^32^P end-labeled aptamer. Increasing amounts of SARS-CoV-2 RBD or other proteins were diluted in the above buffer and were added in amounts that approximately flanked the *K*_D,app_ value (estimated from initial experiments) for the aptamer. After 10 min at room temperature, the reactions were applied to a 25 mm nitrocellulose disk (0.45 µm pore, Protran BA 85, Whatman™) pre-soaked in filter wash buffer (25 mM Tris-HCl pH 7.5, 10 mM KCl). The filter was washed under vacuum with 5 mL of wash buffer at a flow rate of ~1 mL/sec. Filters were then counted in a scintillation counter. A plot of bound aptamer vs. protein concentration was fit to the following equation for ligand binding, one-site saturation in SigmaPlot in order to determine the *K*_D,app_: y = B_max_(x)/(*K*_D_+x) where x is the concentration of protein, and y is the amount of bound aptamer. 

#### 2.2.5. Competition Binding Assays

In total, 10 nM 5′ ^32^P end-labeled aptamer was incubated at room temperature in PBS with various amounts of excess unlabeled competitor at 0-, 1-, 2-, 4-, 8-, or 16-fold excess over radiolabeled labeled aptamer. SARS-CoV-2 RBD or S1 protein was added to a final concentration of 10 nM. The total volume was 20 µL (in PBS). Incubations were continued for 1 h at room temperature. In competition reactions with the human ACE2 protein, the radiolabeled aptamer was mixed with ACE2 prior to the addition of SARS-CoV-2 RBD. Assays for measuring the binding of ACE2 to aptamers in the absence of RBD or S1 were also performed. The low level of binding to the aptamer in the absence of RBD was subtracted away from the result with radiolabeled aptamer, ACE2, and RBD to produce the final result (see Figure 3). Samples were run over a nitrocellulose filter and washed and quantified as described above.

#### 2.2.6. Dissociation Constant (*k*_off_) and Half-Life (t_1/2_) Determinations 

For this, 5 nM (final concentration) 5′ ^32^P end-labeled R8–9 aptamer was incubated for 10 min at room temperature in 90 µL PBS with 5 nM (final concentration) SARS-CoV-2 RBD, S1, or Trimer (as indicated). Unlabeled R8–9 aptamer was then added in a volume of 10 µL of PBS such that the final concentrations of unlabeled R8–9 were 125 nM (25-fold excess over labeled aptamer). For the SARS-CoV-2 Trimer, the concentration of unlabeled R8–9 was increased to 250 nM due to each trimer having 3 binding sites for the aptamer. A total of 10 µL aliquots were removed and filtered over nitrocellulose (see above) at “0”, 10, 20, 40, 60, 80, 100, and 120 min, or as indicated. Note that the time “0” was removed immediately upon the addition of unlabeled aptamer, but it is not a true time 0 as a few seconds passed before the material was filtered over nitrocellulose. Producing a time 0 sample by filtering the material prior to unlabeled aptamer addition produced results that were sometimes inconsistent with the remaining time points and produced experimental fits (see below) that were less accurate (based on r^2^ values). A background control was prepared by mixing 5 nM 5′ ^32^P end-labeled R8–9 aptamer and 125 nM unlabeled aptamer in 9 ul of PBS, then adding 1 uL of SARS-CoV-2 RBD protein (final concentration 5 nM) and incubated for 10 min before processing. The dissociation constant was determined by fitting the data from a plot of aptamer bound to the filter vs. time, to an equation for a single 2-parameter exponential decay in SigmaPlot: y = ae^-bx^, where b is the dissociation constant (*k*_off_ in this case). The *t*_1/2_ value was determined from *k*_off_ using the following equation: *t*_1/2_ = 0.69/*k*_off_. 

#### 2.2.7. Binding Inhibitions Analysis

The ability of aptamers to block the association of SARS-CoV-2 RBD with ACE2 was measured with the cPass™ SARS-CoV-2 Neutralization Antibody Detection kit (GenScript^®^). For comparison, neutralizing monoclonal antibody (GenScript^®^, clone ID: 6D11F2) was also used. The manufacturer’s suggested protocol for the kit was followed. Positive and negative controls were provided by the manufacturer. This protocol included an initial 30 min binding step with RBD and aptamer or antibody, followed by a 15 min incubation of the material with attached ACE2. 

## 3. Results

### 3.1. Selection of FANA Aptamers against the Spike Receptor Binding Domain (RBD) 

**Figure 1:**
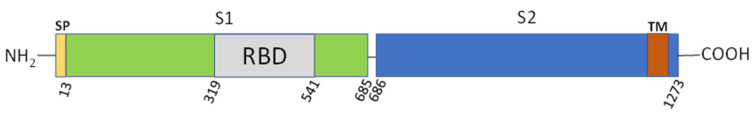
Structure of SARS-CoV-2 spike protein. The spike (S) protein has two major domains, subunit 1 (S1) and subunit 2 (S2). The receptor-binding domain (RBD) that binds to ACE2 is amino acid Arg319-Phe541 of the S1 domain. A 13 amino acid signal peptide (SP) is present at the start of the amino terminus, while the transmembrane domain (TM) is located near the C-terminus (amino acids 1214–1234). The numbering was taken from [44], and a more detailed representation can be found in that reference.

**Figure 2:**
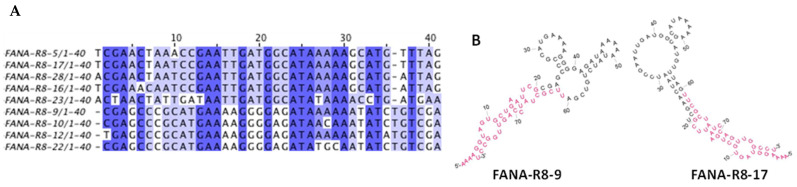
Sequence alignment using the MAFFT program for the random region (nucleotides 1–40) of recovered aptamers in the FANA-R8–17 and FANA-R8–9 lineages: (**A**) FANA-R8–5 to FANA-R8–23 are from the lineage containing FANA-R8–17, and FANA-R8–9 to FANA-R8–22 are from the FANA-R8–9 lineage. The fixed primer regions 5’-AAAAGGTAGTGCTGAATTCG-3′ at the 5′ end and 5′-UUCGCUAUCCAGUUGGCCU-3′ at the 3′ end are not shown in the alignment; (**B**) RNAfold program predicted structures of FANA-R8–9 and 17. Folded aptamers include primer regions. Red nucleotides are from the fixed primer regions at the 5′ and 3′ ends, while black nucleotides were derived from the random region of the starting material.

Aptamers were produced by a modified SELEX approach using mutated enzymes capable of converting between DNA and FANA [36]. The RBD domain of the (SARS-CoV-2) S protein was chosen as the target because aptamers that directly block S protein-ACE2 receptor interactions were desired, rather than those that bind S protein in other domains that may be less likely to block receptor binding. The 223 amino acid RBD (amino acid 319-541), comprises just a small portion of the S protein (1273 amino acids) (Figure 1). It is part of the S1 subunit (amino acids 14-685) present on the outside of the viral envelope [43,44]. The C-terminal His-tagged RBD domain was attached to magnetic beads for the selection process (see Materials and Methods). A total of 28 sequences were recovered from a limited number of clones after 8 rounds of selection. The recovered sequences were organized by sequence similarity into clusters using multiple alignment using fast Fourier transform (MAFFT) [45]. Sequences (7 total) from different clusters with diverse structures based on RNAfold analysis [46] were chosen for further testing. The FANA aptamer sequences are named based on the SELEX round (i.e., R8), and the number of the particular sequence clone. The two clusters containing the strongest RBD binding sequences (see Table 1) are represented by FANA-R8–9 and FANA-R8–17. These sequences are aligned with other recovered sequences from the same clusters (Figure 2A) and the predicted structures of FANA-R8–9 and FANA-R8–17 are shown in Figure 2B. The other sequences from these clusters had similar predicted structures. Aptamers from other clusters in Table 1 (FANA-R8–3, FANA-R8–7, and FANA-R8–15) that bound less tightly also had different predicted structures (data not shown). Filter binding assays were used for measuring the apparent equilibrium dissociation constants (*K*_D,app_) to the RBD protein and the larger S1 portion of the SARS-CoV-2 spike protein (Val16-Arg685). All 7 tested FANA sequences bound to the RBD and S1 protein with ~25-fold of greater binding affinity than the starting material (FANA-ST, Table 1). Aptamers FANA-R8–9, the closely related FANA-R8–22, and FANA-R8–17 bound the strongest, and FANA-R8–9 was chosen for further testing. This aptamer bound both the S1 protein and S protein trimer (a soluble form of the S trimer that is present on viral membranes) modestly more tightly than the RBD. A version of S1 without the His-tag bound with approximately the same affinity to FANA-R8–9 as tagged protein indicating that the His-tag played no role in aptamer binding. FANA-R8–9 was also tested for binding the Delta variant RBD protein. This protein differs from the Wuhan strain within the RBD domain at two positions, Lys452Arg and Thr478Lys. Interestingly, FANA-R8 bound about 10-fold better to this protein than the Wuhan strain RBD it was selected to. A similar phenomenon was observed for a DNA aptamer selected by the Wuhan RBD, which bound better to the UK variant [47]. The binding of FANA-R8–9 to the S1 protein from SARS-CoV-1 was also tested (Table 1). The spike proteins from these two viruses, which both use ACE2 as a receptor, are ~76% identical at the amino acid level and ~74% identical in the RBD domain [48]. The several-fold lower binding to S1 from SARS-CoV-1 demonstrates the high specificity of FANA-R8–9. The binding of RBD and S1 to DNA aptamer selected for RBD binding (CoV2-RBD-1C) was also tested. Aptamer CoV2-RBD-1C has a reported *K*_D_ for RBD of 5 nM, which suggests modestly tighter binding to RBD than FANA-R8–9 [49]. This aptamer bound weakly to our RBD construct but did bind to the S1 protein, albeit with lower affinity than the FANA aptamers. Differences between the published results and ours may reflect the different affinity measurement techniques or different protein constructs (see Discussion). 

**Figure 3:**
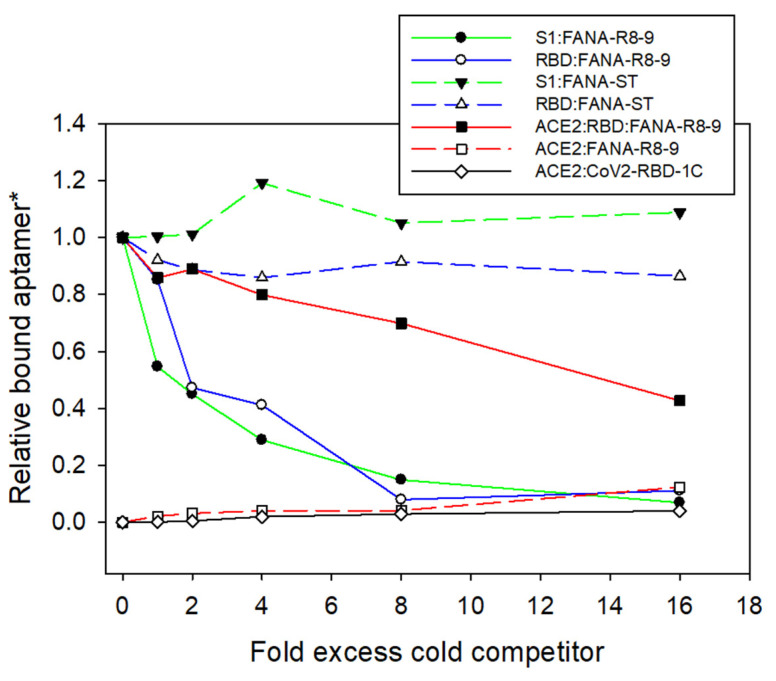
Competition binding assay with SARS-CoV-2 RBD and S1 proteins. Samples contained 10 nM of 5′-^32^P end-labeled FANA-R8–9 aptamer and 10 nM of either RBD or S1 proteins (see below). Cold competitor (FANA-R8–9, FANA-ST (starting material for SELEX), or ACE2 protein) was added at 0, 1, 2, 4, 8, and 16-fold excess over labeled FANA-R8–9. RBD and S1 were omitted from the ACE2:FANA-R8–9 and ACE2:CoV2-RBD-1C (51 nt DNA aptamer). * All values are relative to the value for no competitor with RBD:FANA-R8–9 or S1:FANA-R8–9 (for S1 samples only).

Aptamer FANA-R8–9 was used in competition binding and off-rate analysis experiments. As expected, non-labeled FANA-R8–9 was able to compete with radio-labeled aptamer for binding to RBD or S1 (Figure 3). However, non-labeled FANA-ST (starting material for SELEX selections) was unable to displace any FANA-R8–9 aptamer, even when added at 16-fold greater amounts. This confirms that FANA-R8–9 binds to RBD much more tightly than the starting material. In contrast, ACE2 protein was able to compete with FANA-R8–9 for binding to RBD. However, 2-fold excess cold FANA-R8–9 was as effective as 16-fold excess ACE2 in the competition. This indicates that FANA-R8–9 binds better to RBD than ACE2. ACE2 was also tested for binding to FANA-R8–9 and the CoV2-RBD-1C DNA aptamer [49]. Weak binding to ACE2 was detected for both aptamers, with twofold greater binding to FANA-R8–9 (Figure 3).

**Figure 4:**
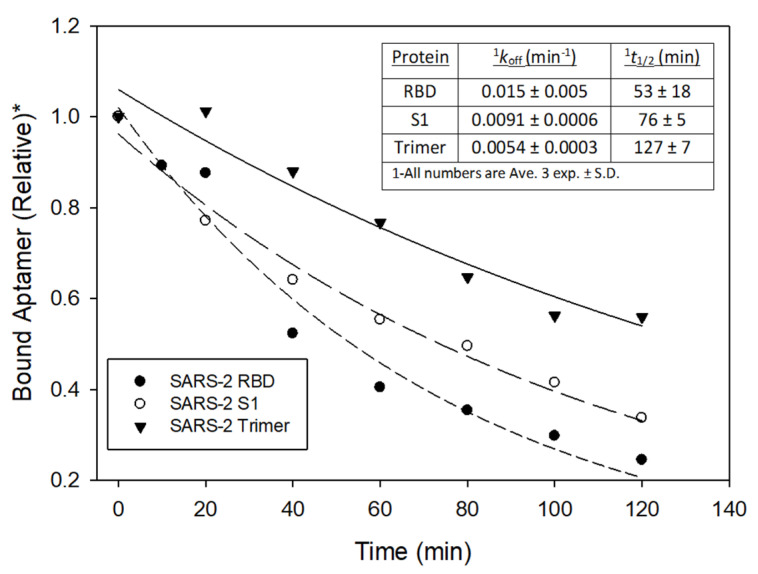
Example of an off-rate analysis of FANA-R8–9 from SARS-CoV-2 RBD, S1, and trimer proteins. Experiments to examine the dissociation of aptamer from various proteins were conducted as described in Materials and Methods. Data was fit to a curve for single parameter exponential decay to calculate off-rate (*k*_off_) and half-life (t_1/2_). The experiment was repeated 3 times to yield *k*_off_ and t_1/2_ values shown in the insert table. The FANA-ST starting material bound very weakly in this assay with no significant level of bound material (using RBD) being measurable at the 20 min time point. * Values are relative to the value for bound material at time 0.

Off-rate analysis showed that FANA-R8–9 dissociated from RBD with a half-life of 53 ± 18 min (Ave. 3 exp. ± S.D., Figure 4), demonstrating stable binding. Binding appeared to be more stable with SARS-CoV-2 S1 protein (76 ± 5) and the S protein trimer (127 ± 7 min). While the increase was not statistically significant for the S1 protein, it was for the S protein. More stable binding may be due to additional stabilizing contacts being available on the larger S protein. Another possibility for the S protein trimer is that there is a proximity effect since 3 RBD binding sites for the aptamer are presumably close together and an aptamer that dissociated from a site may quickly bind to a neighboring site. 

### 3.2. Functional Assessment of RBD-Binding Aptamers 

**Figure 5:**
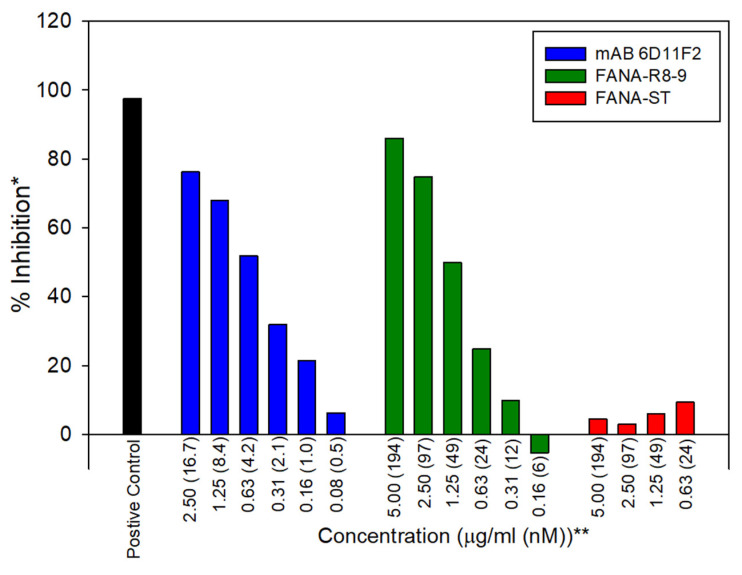
ELISA assay to test the ability of antibodies and aptamers to block ACE2 binding to the RBD domain. Neutralizing RBD antibody (GenScript 6D11F2) was compared with FANA-R8–9 and FANA-ST (starting material) in an ACE2 ELISA assay (GenScript). * Percent inhibition was calculated based on positive and negative controls supplied by the manufacturer. The negative control produced a value of “0” in the assay and is not shown. See Materials and Methods for more details. ** Values are given in both µg/mL and nM. Note that each antibody contains two binding sites. The experiment was repeated with similar results. See text for more details.

To measure the ability of aptamers to block the binding of the RBD to the ACE2 receptor, an ACE2 ELISA was used (Figure 5). Aptamers were compared with an anti-SARS-CoV-2 RBD-neutralizing antibody (GenScript^®^ clone ID: 6D11F2) and FANA-ST. On a weight basis (Figure 5, see μg/mL amounts on X-axis), antibody 6D11F2 and FANA-R8–9 (as well as FANA-R8–22 (data not shown)) showed similar ability to block ACE2 binding (IC_50_ ~0.6 µg/mL for the antibody and 1.30 ± 0.18 µg/mL (ave. 3 exp. ± S.D.) for FANA-R8–9), while aptamer FANA-R8–17 was ~ threefold weaker (data not shown), and FANA-ST showed no significant blocking of ACE2 binding. On a per molecule basis, the antibody was more effective as an inhibitor (Figure 5, see “nM” amounts on X-axis). Considering that antibodies have two target binding sites vs. one on the aptamer, the antibody was about threefold better using this criterion. 

**Figure 6:**
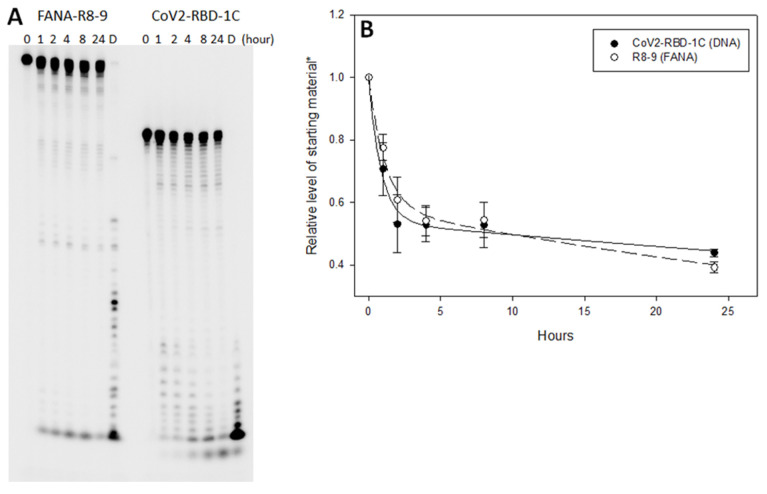
(**A**,**B**). Aptamer stability assay. 100 nM of radiolabeled FANA-R8–9 (79 nts) and DNA aptamer CoV2-RBD-1C (51 nts (46)) were incubated in 200 uL of D-MEM complete + 10% FBS, and 1% penicillin/streptomycin) at 37 °C. Twenty ul aliquots were removed at 0, 1, 2, 4, 8, and 24 h time points and run on a 10% PAGE denaturing gel. Lane ‘D’, a 20 uL aliquot was digested with DNaseI for 30 min at 37 °C as a control; (**B**) quantification of products. Gels were visualized and quantified using a phosphorimager. The level of full-length undegraded material was measured at each time point. The graph shows averages from three independent experiments with error bars representing the standard deviations. * Values were relative to the amount of material at time 0.

FANA-R8–9 was also tested for stability in serum-containing cell culture media (Figure 6A). Both FANA-R8–9 and CoV2-RBD-1C DNA aptamer [49] remained intact for several hours and demonstrated similar decay rates (Figure 6B). The slower rate of decay between 4 and 24 h may result from decreased activity of the degrading enzymes in the media. 

## 4. Discussion

This report describes the production of aptamers that can bind to and block the binding of the SARS-CoV-2 RBD to ACE2. The aptamers are unique, as they are made from FANA XNA, as opposed to previous DNA aptamer to the SARS-CoV-2 S protein. Binding, based on *K*_D,app_ analysis was comparable to previously reported DNA aptamers [47,49,50,51,52]. The aptamers were stable for several hours in cell culture media but did break down at a rate comparable to the tested DNA aptamer (Figure 6). 

Interestingly, a previously reported DNA aptamer (CoV2-RBD-1C) that bound with a *K*_D_ of 5.8 ± 0.8 nM to RBD [49] did not bind strongly to the RBD in our system, although it did show binding to the S1 domain protein, albeit at a lower level than the FANA aptamers (Table 1). The RBD used for binding tests in our experiments was the same as the protein used for selection and included a C-terminal His-tag. Binding was also measured in solution using nitrocellulose filters. The CoV2-RBD-1C aptamer was measured using RBD attached to nickel beads. It is possible that the free His-tag (as opposed to the tag sequestered on beads) in our measurements interfered with binding. The S1 protein used in our measurements also contained a His-tag, but it is further away from the RBD domain due to the larger size of the protein. Other DNA aptamers to RBD have also been reported. Most report binding in the same low nM range, as the FANA aptamers described here [47,49,51] (https://www.basepairbio.com/COVID19/, accessed on 1 July 2021). This is in the same range as the reported interaction between ACE2 and the SARS-CoV-2 S protein (14.7 nM) and considerably tighter than SARS-CoV-1 S protein binding to ACE2 (325.8 nM) [53]. Therefore, it would be expected that these aptamers should be good competitors for ACE2 binding. In agreement with this observation, FANA-R8–9 was about as effective on a per weight basis as the neutralizing RBD-specific antibody used in this analysis (Figure 5). As there are numerous variations in the type of aptamers that can be generated with different XNAs [54,55], perhaps those that bind even more tightly can be obtained in the future. 

The FANA-R8–9 and other aptamers (Table 1) bound with low nM affinity to RBD, while previous FANA aptamers isolated in this lab to HIV reverse transcriptase (RT) and integrase (IN) bound with low pM affinity, ~1000-fold tighter [41,42]. One reason for this is RT and IN are both natural nucleic acid binding proteins and already bind tightly to specific nucleic acids. It is more of a challenge to recover strong binding aptamers to proteins that do not naturally bind nucleic acids. However, this is not always the case. Aptamers to thrombin, for example, can bind with pM affinity and modified aptamer to VEGF, which is the target for aptamer therapy for macular degeneration, also show pM binding [56,57,58]. Several aptamers made using slow off-rate modified aptamers (SOMAs) technology that includes the addition of hydrophobic groups to nucleic acids bind tightly to targets, even those that are not natural nucleic acid-binding proteins [59]. Still, making aptamers is a “hit-or-miss” proposition, and there are no guarantees that aptamers that can bind more tightly than those reported here or by others can be found. It was notable that despite the modest low nM *K*_D_’s of the FANA aptamers (Table 1), the observed off-rates were indicative of highly stable binding, especially for the more natural trimeric S protein (Figure 4). Stable binding is likely a better predictor of potential therapeutic effect than a lower binding affinity [60]. 

Finally, we have not yet tested the FANA aptamer in virus neutralization assays. Aptamers that block interactions with the receptor, such as neutralizing antibodies, have the advantage of not having to enter cells to be effective. While DNA aptamers that bind the SARS-CoV-2 RBD have been shown to block virus infection [51,52], a recent report indicates that a DNA aptamer that binds to S1 but not in the RBD region can neutralize virus [50]. Interestingly, this aptamer did not appear to block virus binding to the ACE2 receptor. This suggests that even those aptamers that do not directly block binding may be able to inhibit replication. 

## Figures and Tables

**Table 1:** Apparent equilibrium dissociation constants (*K*_D_,app) for tested FANA aptamers.

^a^ Aptamer	^b^ RBD *K*_D,app_ (nM)	^c^ S1 *K*_D.app_ (nM)	S1 (No His Tag) *K*_D,app_ (nM)	^d^ S Protein Timer *K*_D,app_ (nM)
^e^ FANA-ST	^f^ 1695 ± 754			
FANA-R8–3	68.1 ± 16.9	46.4 ± 2.8		
FANA-R8–5	26.4	16.6		
FANA-R8–7	62.8 ± 8.4	31.2 ± 11.7		
FANA-R8–9	23.5 ± 4.6	14.7 ± 3.9	29.6 ± 3.9	14.4 ± 4.6
FANA-R8–9	1.4 ± 0.4 (Delta Variant) ^g^			
^h^ FANA-R8–9 (D-MEM+10% FBS)	19.6 ± 6.2			
FANA-R8–15	43.3 ± 0.2	27.7 ± 5.3		
FANA-R8–17	29.5 ± 6.5	19.1 ± 7.5		
FANA-R8–22	21.3	13.3		
^i^ S1-SARS-CoV-1		259 ± 161		
^j^ CoV2-RBD-1C	872 ± 359	105 ± 34		

a—Aptamer sequences. Only the ~40 nt random regions of the ~79 nt aptamers are shown. In the full aptamer, this would be flanked by 5′-AAAAGGTAGTGCTGAATTCG-3′ at the 5′ end and, and 5′-UUCGCUAUCCAGUUGGCCU-3′ at the 3′ end, (see Methods)): FANA-ST: 5′-NNNNNNNNNNNNNNNNNNNNNNNNNNNNNNNNNNNNNNNN-3′. FANA-R8–3: 5′-GTCGCGATTAACATTAAACCGCATAAAAAGGGTGGCCGGA-3′. FANA-R8–5: 5′-TCGAACTAAACCGAATTGATGGCATAAAAAGCATGTTTAG-3′. FANA-R8–7: 5′-ATTCTCGATTGATGGCATAAAAAGCATAGAATCGCAAGCA-3′. FANA-R8–9: 5′-CGAGCCCGCATGAAAAGGGGAGATAAAAAATATCTGTCGA-3′. FANA-R8–15: 5′-GGAGCTCGAACAGATGGGATAAAAAGCATAGCTCACCAAT-3′. FANA-R8–17: 5′-TCGAACTAATCCGAATTGATGGCATAAAAAGCATGTTTAG-3′. FANA-R8–22: 5′-CGAGCCCGCATGAAAAGGGGAGATATGCAATATCTGTCGA-3′. b—“RBD”: Receptor binding domain of SARS-CoV-2 spike protein (Wuhan strain unless otherwise stated) with C-terminal His-tag (see Figure 1). c—“S1”: S1 portion of SARS-CoV-2 spike protein (Wuhan strain) with C-terminal His-tag (see Figure 1). d—Modified S protein trimer from SARS-CoV-2 (aa 1–1208), furin cleavage site removed, C-terminal His-tagged. e—FANA-ST: starting material for the SELEX procedure (see above under “a”). f—*K*_D,app_ values were determined using nitrocellulose filter binding in PBS buffer unless otherwise stated. Results are averages of 2–3 experiment ± standard deviation (for experiments with 3 determinations only). g—RBD from the Delta variant differs from Wuhan strain RBD at two amino acids: Lys452Arg, Thr478Lys. h—Assays were performed in cell culture media: D-MEM + 10% fetal bovine serum (FBS). i—Binding of S1 protein from SARS-CoV-1 (2003 virus) to FANA-R8–9. j—DNA aptamer from [49].
